# Exploration of biocompatible AIEgens from natural resources†
†Electronic supplementary information (ESI) available: NMR, DLS results, PL spectra, cell viability, cell imaging, photostability, of BBR chloride. See DOI: 10.1039/c8sc01635f


**DOI:** 10.1039/c8sc01635f

**Published:** 2018-06-29

**Authors:** Yuan Gu, Zheng Zhao, Huifang Su, Pengfei Zhang, Junkai Liu, Guangle Niu, Shiwu Li, Zhaoyang Wang, Ryan T. K. Kwok, Xin-Long Ni, Jingzhi Sun, Anjun Qin, Jacky W. Y. Lam, Ben Zhong Tang

**Affiliations:** a Department of Chemistry , Department of Chemical and Biological Engineering , Hong Kong Branch of Chinese National Engineering Research Center for Tissue Restoration and Reconstruction , Institute for Advanced Study , Division of Biomedical Engineering , Division of Life Science and State Key Laboratory of Molecular Neuroscience , The Hong Kong University of Science and Technology (HKUST) , Clear Water Bay , Kowloon , China . Email: tangbenz@ust.hk; b HKUST – Shenzhen Research Institute , No. 9 Yuexing 1st RD, South Area Hi-tech Park, Nanshan , Shenzhen 518057 , China; c NSFC Center for Luminescence from Molecular Aggregate , SCUT-HKUST Joint Research Laboratory , State Key Laboratory of Luminescent Materials and Devices , South China University of Technology , Guangzhou 510640 , China; d MOE Key Laboratory of Macromolecular Synthesis and Functionalization , Department of Polymer Science and Engineering , Zhejiang University , Hangzhou 310027 , China; e MOE Key Laboratory of Macrocyclic and Supramolecular Chemistry of Guizhou Province , Guizhou University , Guiyang , Guizhou 550025 , China

## Abstract

Luminogens with aggregation-induced emission (AIEgens) characteristics have been well developed and applied in various areas such as bio-imaging, theranostics, organic photoelectronics and chemo/bio sensors. However, most of the reported AIEgens suffer from the disadvantages of complex organic synthesis and high cost, as well as being environmentally unfriendly and hard to degrade, which have largely limited their real applications. In this work, we discovered berberine chloride, a natural isoquinoline alkaloid isolated from Chinese herbal plants, as an unconventional rotor-free AIEgen with bright solid-state emission and water-soluble characteristics. Single crystal structure analysis and optical property, viscosity, and host–guest interaction studies suggested that intramolecular vibration and twisted intramolecular charge transfer were responsible for the AIE phenomenon of berberine chloride. Moreover, berberine chloride was biocompatible and could specifically target lipid droplets in a fluorescence turn-on and wash-free manner, demonstrating the great potential of natural products as promising AIE probes.

## Abstract 2

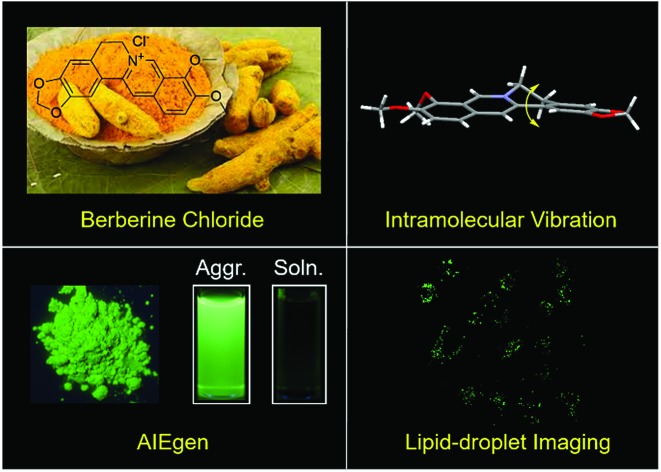
In this contribution, we propose natural resources as a new source to acquire biocompatible AIEgens.

## Introduction

Light is indispensable to the survival of human beings. Driven by curiosity and the key role of light in human life, people have never stopped seeking ideal luminogens and exploring the essence of luminescence. Indeed, the study of luminescent materials has not only deepened our understanding of the underlying mechanism of the luminescence process, but has also created many advanced technologies that are changing our lives, including illumination, fiber-optic communication and optical diagnosis.^1^–^3^

Traditional planar luminogens usually exhibit bright emission in dilute solutions but emit dimly or even no light at all when aggregated or in the solid state, which has been known as the aggregation-caused quenching (ACQ) effect.^4^ This ACQ effect has greatly prevented them from realizing their full potential in many practical applications.^5^ In 2001, Tang and his coworkers found a class of compounds exhibiting a phenomenon opposite to ACQ: they showed negligible emission in dilute solutions but enhanced fluorescence in the aggregated or solid state, which they termed aggregation-induced emission (AIE).^6^ The discovery of AIE has elegantly solved the ACQ problem of traditional luminogens. A further mechanism study revealed that restriction of intramolecular motion (RIM) including rotation and vibration played a key role in the AIE phenomenon.^7^ Guided by the RIM principle, various AIEgens have been designed and synthesized and applied in different areas such as bio-imaging, chemical sensors and optoelectronics.^8^

So far, almost all the AIEgens have been produced by organic synthesis. Despite the advantages of the diversity and colour-tunability of these man-made luminogens, they are commonly associated with the disadvantages of complex organic synthesis and high cost, as well as being environmentally unfriendly and hard to degrade, which actually limit their practical applications. Furthermore, considering that biological research studies are usually conducted in aqueous media, water-soluble AIEgens thus hold an intrinsic advantage.8b,c Therefore, the exploration of new sources to obtain large-scale, biocompatible, water-soluble, and degradable AIEgens is of big significance. On the other hand, China has a long history of studying Chinese herbs.^9^ And the active ingredients of many herbal formulations, such as artemisinin, berberine and curcumin, can be facilely and efficiently obtained with the development of large-scale planting and modern extraction techniques.^10^–^13^ Although these natural products have played significant roles in treating some common diseases such as malaria, dysentery and inflammation, their luminescent properties have scarcely been investigated, which not only are fundamentally interesting, but also facilitate the development of potential candidates for advanced theranostics.^14^,^15^

Here, we reported Berberine (BBR) chloride, an isoquinoline alkaloid isolated from many herbal plants such as *Hydrastis canadensis*, Cortex Phellodendri and Rhizoma coptidis, as a unique natural aggregation-induced emission luminogen (AIEgen).^16^–^18^ Although BBR chloride has been widely studied and found to exhibit multiple biological and pharmacological properties including inhibiting acetylcholinesterase and reducing cholesterol and glucose, as well as immunomodulatory, antimicrobial, and anti-inflammatory properties,^18^,^19^ until now, its AIE characteristics have scarcely been reported. Additionally, unlike the typical AIEgens with a propeller structure and rotors such as tetraphenylethylene (TPE), BBR chloride is an unconventional rotor-free AIEgen.20*a* The AIE properties and mechanism of BBR chloride have been systematically investigated by UV-vis and PL spectroscopy, single crystal structure analysis, PL spectral changes with host–guest interaction, and viscosity and temperature variation. In addition, BBR chloride exhibited superior selectivity for lipid droplet imaging at cellular and living liver tissue levels. This work not only demonstrates a new biocompatible and rotor-free AIEgen with selective lipid droplet imaging, but also provides a new source to acquire more natural AIEgens with obvious advantages over artificial luminogens.

## Results and discussion

### Aggregation-induced emission

**Fig. 1:**
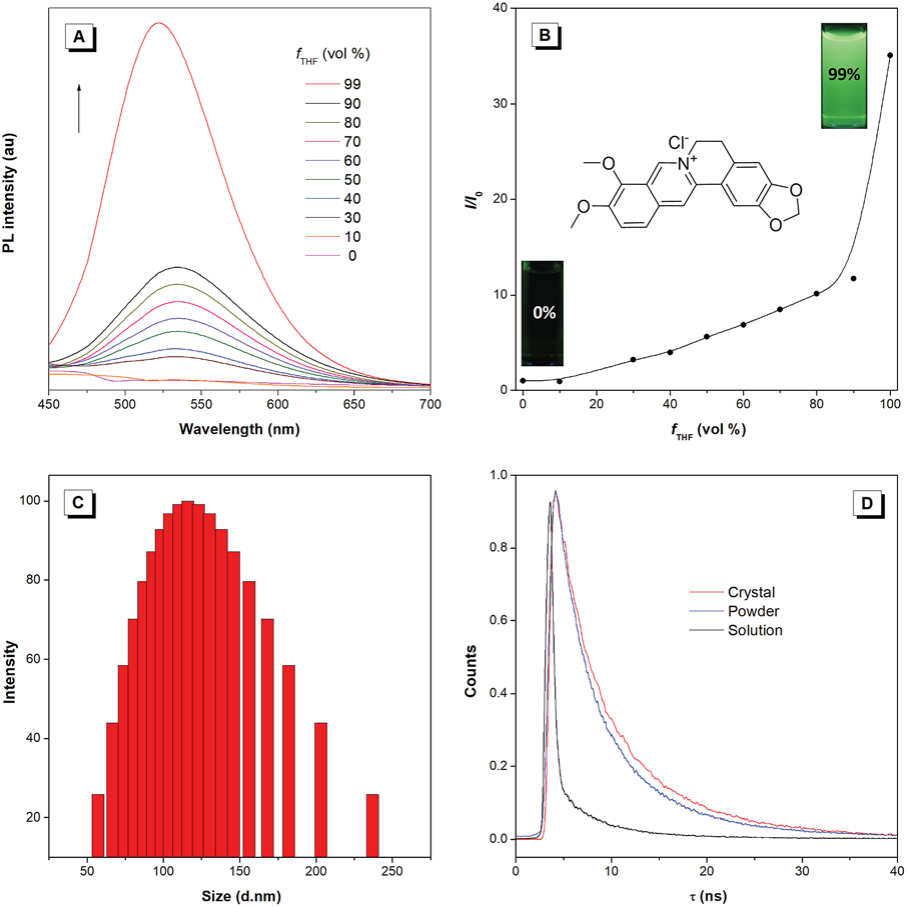
(A) PL spectra of BBR chloride in water and water/THF mixtures with different THF fractions (*f*_T_). Slit width: 5 nm. (B) Plots of the *I*/*I*_0_ value *versus f*_T_. (C) DLS result of BBR chloride in a THF/water mixture (*f*_THF_ = 90%). Concentration: 10 μM. Excitation wavelength: 405 nm. (D) Time-resolved emission decay curves of BBR chloride in aqueous solution, powder and crystal states.

BBR chloride is a water-soluble and commercially available molecule with a donor–acceptor structure. The studied BBR chloride was purchased from Meyer Co., Ltd., whose structure and purity were confirmed by ^1^H NMR and HPLC (Fig. S1†). The AIE characteristics of BBR chloride were investigated by studying its photoluminescence (PL) behaviours in water and water/tetrahydrofuran (THF) mixtures. BBR chloride shows no emission in dilute water solution but enhanced emission with increasing the THF fraction (*f*_T_) from 10% to 99% (Fig. 1A and B). Dynamic light scattering (DLS) results indicate that aggregates are formed following the addition of THF (Fig. 1C and S2†). These results indicate that BBR chloride was AIE active and the enhanced emission of BBR chloride in water/THF mixtures originated from its aggregation in poor solvents. The concentration effect also supports the AIE feature of BBR chloride since much enhanced emission is exhibited at a high concentration of BBR chloride (Fig. S3†). The photoluminescence quantum yields (PLQYs) of BBR chloride in water solution, nanosuspension, powder and crystal states were determined using an integrating sphere, giving the corresponding PLQYs of 0.2%, 2.9%, 12%, and 15%, respectively, which are in good accordance with the AIE characteristics of BBR chloride. To better understand the AIE characteristics of BBR chloride, we measured its emission lifetimes and investigated its radiative and non-radiative decay processes in solution and solid states (Fig. 1D and Table S1†). The results indicate that the lifetime of the powder (4.86 ns) and crystal (7.93 ns) of BBR chloride is much higher than that of the solution (0.68 ns), which matches with its AIE properties.20b,c Additionally, from the solution to the solid state, the radiative decay rate (*k*_r,soln_ = 0.022 × 10^8^ s^–1^, *k*_r,powder_ = 0.24 × 10^8^ s^–1^, *k*_r,crystal_ = 0.19 × 10^8^ s^–1^) of BBR chloride increases around 9 times while its non-radiative decay rate (*k*_nr,soln_ = 14.69 × 10^8^ s^–1^, *k*_nr,powder_ = 1.82 × 10^8^, *k*_nr,crystal_ = 1.08 × 10^8^ s^–1^) decreases about 14 times, leading to its much enhanced fluorescence in the solid state. The decrease of the *k*_nr_ is possibly due to the more rigid environment in the crystal state, which can restrict the molecular motion more efficiently.

### Single crystal packing

**Fig. 2:**
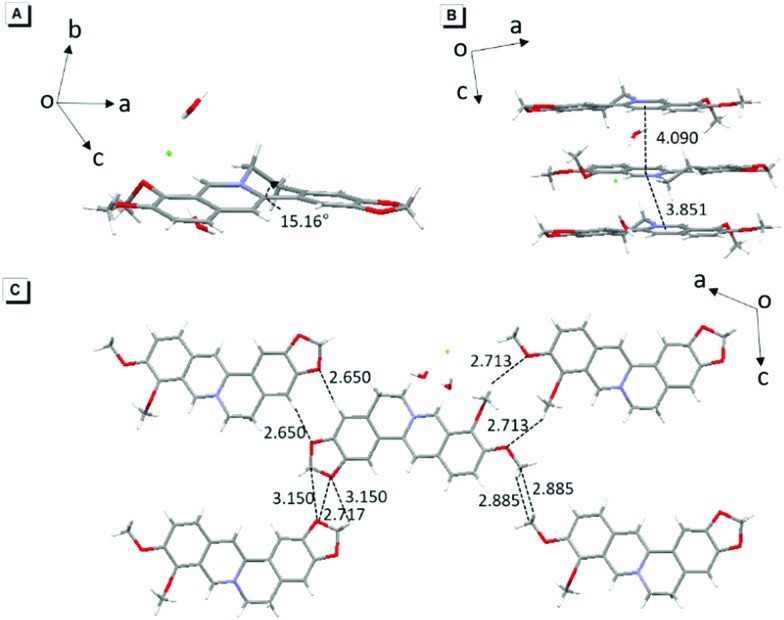
(A) Intramolecular torsional angle in BBR chloride. (B) Intermolecular π–π distance of two adjacent molecules in BBR chloride. (C) Intermolecular C–H···O, C–H···C, C···O, and O···O interactions in BBR chloride.

The single-crystal structure of BBR chloride will help in understanding its photophysical behavior.^21^ As shown in Fig. 2, BBR chloride adopts a non-planar conformation in its crystal with a twisted angle of 15.16° between the electron-donating phenyl group and the isoquinoline group, suggesting that intramolecular vibronic motion behaviour and the twisted intramolecular charge transfer (TICT) effect^22^ possibly exist (Fig. 2A). Additionally, the adjacent BBR chloride molecules are aligned in a nearly parallel manner with intermolecular distances of 3.851 Å and 4.090 Å, exceeding the typical π–π stacking distance (3.5 Å) that usually quenches the fluorescence. Moreover, multiple intermolecular C–H···O, C–H···C, C···O, and O···O interactions with distances in the range of 2.6–3.1 Å were observed (Fig. 2C), which help rigidify the molecular conformation and make BBR chloride strongly emissive in the crystal state. Therefore, the weak emission of BBR chloride in dilute water solution is possibly ascribed to the active intramolecular vibration behaviour and/or the TICT effect which open the access to non-radiative decay, while in the aggregates or solid state, the non-radiative decay pathway is largely suppressed, thus resulting in bright fluorescence.

### Mechanism study

**Fig. 3:**
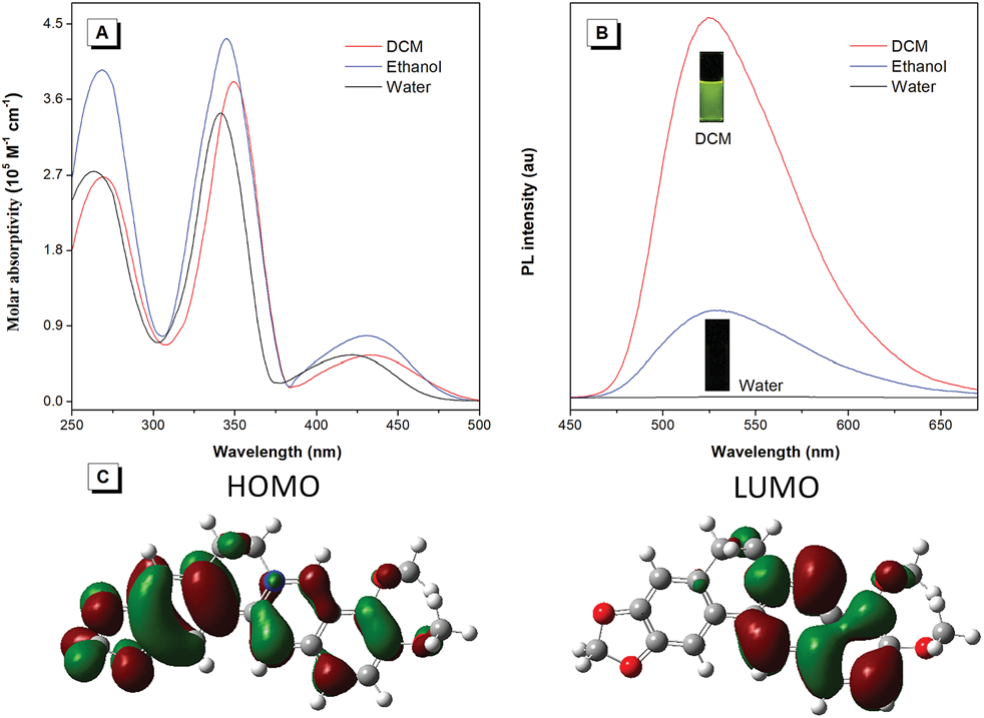
(A) UV-vis spectra of BBR chloride in H_2_O, ethanol, and DCM. (B) PL spectra of BBR chloride in H_2_O, ethanol and DCM. Slit width: 3 nm. (C) Molecular orbital amplitude plots of the HOMO and LUMO energy levels of BBR chloride.

**Fig. 4:**
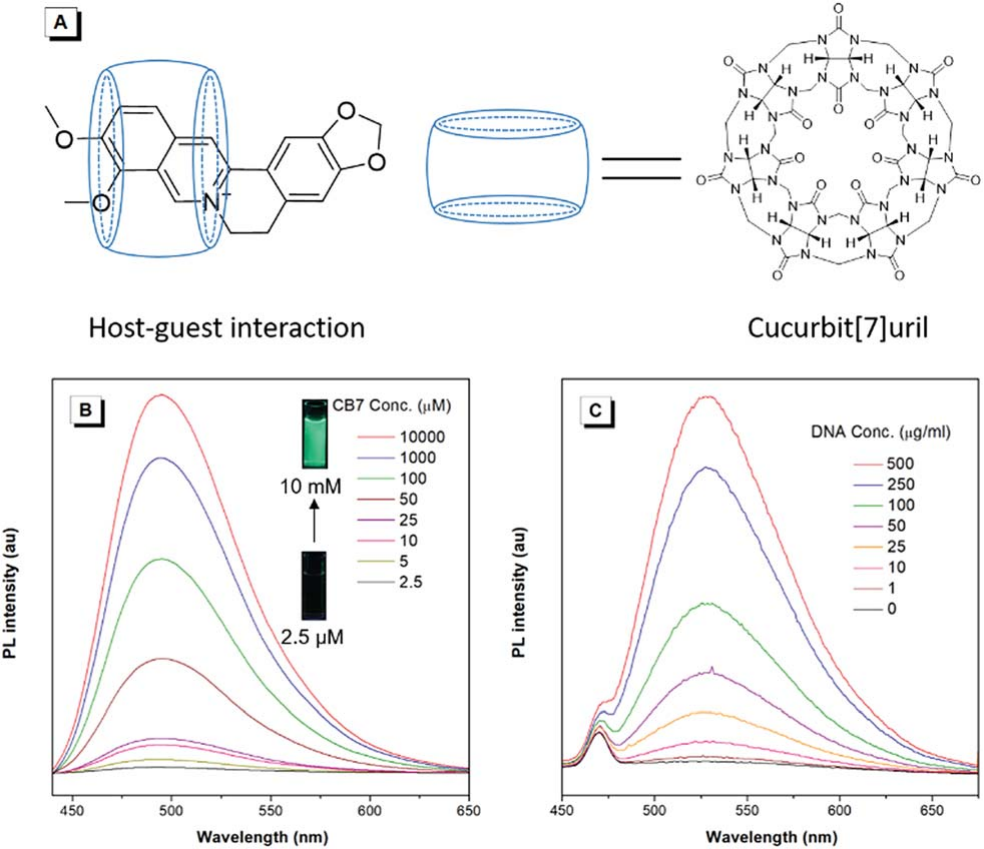
(A) Schematic illustration of host–guest interactions between BBR chloride and cucurbit[7]uril (CB7). (B) PL spectra of BBR chloride in cucurbit[7]uril (CB7) aqueous solution with different concentrations. Slit width: 4 nm. (C) PL spectra of BBR chloride in bovine thymus DNA aqueous solution with different concentrations. Slit width: 5 nm. Excitation wavelength: 405 nm.

To unveil the underlying mechanism of the AIE-active BBR chloride, we studied its photophysical behavior in solvents with different polarities to reveal whether the TICT effect exists. As shown in Fig. 3A, with increasing the solvent polarity, the absorption of BBR chloride is almost unchanged while its fluorescence intensity shows a pronounced decrease, followed by a red shift of the emission maximum from 525 nm to 550 nm (Fig. 3B), suggesting that the TICT effect indeed exists.^22^ In addition, the electron density distribution of the HOMO and LUMO is indicative of a π–π* transition with a degree of charge transfer character (Fig. 3C), which agrees well with the experimental data. To investigate the effect of intramolecular vibrations on the photophysical properties of BBR chloride, we used cucurbit[7]uril (CB7)^23^ to restrict its molecular motion. As expected, CB7 and BBR chloride form a host–guest complex and light up the emission of BBR chloride with a blue-shift of the emission peak from 550 nm to 495 nm (Fig. 4A and B).^24^ This is because BBR chloride can enter the hydrophobic cave of CB7 efficiently, as has been reported previously by Megyesi *et al.*,24*a* which not only restricts the intramolecular vibration of BBR chloride, but also produces a non-polar micro-environment to blue-shift and enhance the emission. It is also worth noting that the TICT effect is sensitive to the molecular conformation change; therefore, the molecular motion and TICT effect coexist mostly in twisted D–A systems.^25^ To further elucidate the role of intramolecular vibration in determining the PL properties, we studied the fluorescence changes of BBR chloride upon viscosity and temperature variation. As shown in Fig. S4,† when adding glycerol into the ethylene glycol solution of BBR chloride to increase the viscosity of the mixture, the emission becomes stronger and stronger due to the suppression of the molecular vibration. Similarly, by decreasing the temperature of the aqueous solution of BBR chloride to freeze the molecular motion, the emission also shows a sharp enhancement. Moreover, BBR chloride can interact with DNA by electrostatic interaction;^26^ consequently, the intramolecular vibration is suppressed, and fluorescence is observed (Fig. 4C). These results suggest that both intramolecular vibration and TICT effect play key roles in the AIE phenomenon of BBR chloride.

### Lipid droplet and living liver tissue imaging

**Fig. 5:**
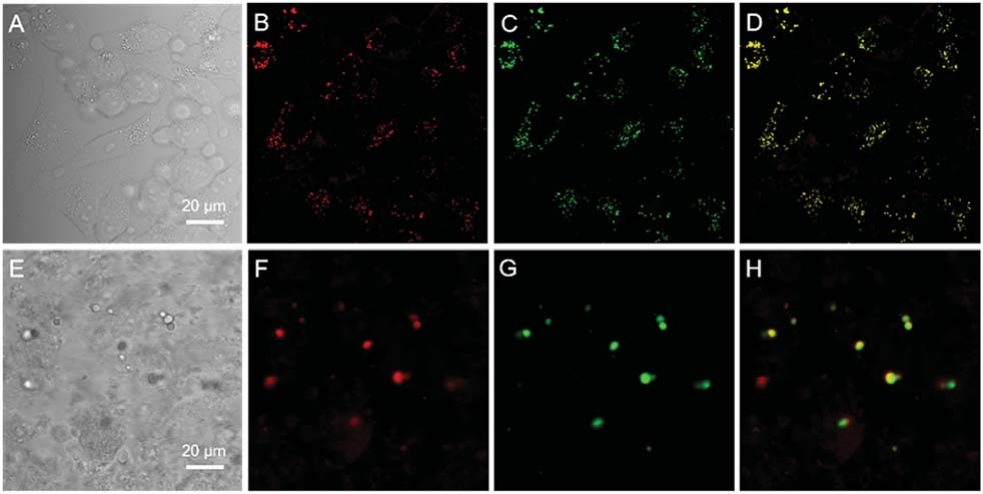
Confocal fluorescence images of HeLa cells and living liver tissue stained with MeOTTMN and berberine chloride. (A) Bright-field image and (B and C) fluorescence images of HeLa cells stained with MeOTTMN (2 μM) (B) and berberine chloride (10 μM) (C) for 30 min. (D) Merged image of panels (B) and (C). (E) Bright-field image and (F and G) fluorescence images of living liver tissue stained with MeOTTMN (4 μM) (F) and berberine chloride (20 μM) (G) for 2 h. (H) Merged image of panels (F) and (G). *λ*_ex_: 488 nm; scale bar = 20 μm.

Since water soluble AIEgens have great potential in wash-free bio-imaging applications,^27^ we thus explored the utilization of BBR chloride in living cell imaging. Before such an exploration, the cytotoxicity of BBR chloride was firstly evaluated using 3-(4,5-dimethyl-2-thiazolyl)-2,5-diphenyltetrazolium bromide (MTT) assay. As shown in Fig. S5,† no significant variation in the cell viability was observed even at a high dye concentration of 20 μM, suggesting the good cellular biocompatibility of BBR chloride. Then, HeLa cells were incubated with BBR chloride, which seemed to be located in the lipid droplets (LDs) of the cell. We then treated the HeLa cells with oleic acid, which can induce significant amounts of neutral lipids in cells.^28^ Clearly, the LDs can be selectively stained with BBR chloride (Fig. 5). To further verify the specificity of BBR chloride in staining LDs, a co-staining experiment was carried out with commercial MeOTTMN dye that targets LDs.^29^ The good overlap (Pearson correlation coefficient: 0.99) demonstrates the superior selectivity of BBR chloride (Fig. 5). It is also worth noting that BBR chloride can selectively stain LDs of other cell types, including A549 and MCF-10A cells (Pearson correlation coefficient: 0.89 for A549 cells; 0.90 for MCF-10A cells), demonstrating that BBR chloride is a promising fluorescent probe for LD staining (Fig. S6†). Photostability is one of the key criteria for evaluating a fluorescent visualizer.^30^ To evaluate the anti-photobleaching capability of BBR chloride, we continuously scanned the HeLa cells and 786-O renal carcinoma cells stained with BBR chloride and green fluorescent protein (GFP), respectively, with laser light. As shown in Fig. S7,† more than 50% of the signal of BBR chloride is retained even after 20 scans, while over 80% of the fluorescence of GFP is lost under the same conditions. Therefore, BBR chloride shows a much higher photostability than green fluorescent protein (GFP). Due to its advantages of low cytotoxicity, high specificity to LDs, wash-free capability and high photostability, we further explored the application of BBR chloride in staining LDs of mice living liver slices. As shown in Fig. 5E–H, after incubation with BBR chloride and MeOTTMN, the lipid droplets of the mice living liver slice exhibit strong green fluorescence of BBR chloride (Pearson correlation coefficient: 0.94). This makes BBR chloride a promising candidate for LD imaging both in cells and in tissue slices, providing a useful tool for tissue slice-based disease diagnosis of lipid droplets.

## Conclusions

In summary, BBR chloride, a natural product isolated from herbal plants has been discovered to be a novel rotor-free AIEgen. In comparison with other reported AIEgens, BBR chloride holds the advantages of water solubility, biocompatibility, and being synthesis free. Single crystal structure analysis indicates that intramolecular vibration and the TICT effect possibly result in its AIE phenomenon, which are further supported by experiments of solvatochromic effect, viscosity effect, temperature effect, host–guest interaction, and electrostatic interaction. Moreover, as a fluorescent probe, BBR chloride can selectively target lipid droplets in cells and in living liver tissue in a fluorescence turn-on fashion by a wash-free method, demonstrating its promising applications in cell and tissue imaging. Therefore, the current work not only demonstrates a biocompatible and rotor-free AIEgen with selective lipid droplet imaging, but also proposes a new source to acquire more natural AIEgens with obvious advantages over artificial luminogens.

## Experimental procedures

### Materials

MeOTTMN was purchased from AIEgen Biotech Co., Ltd. Oleic acid was purchased from Aldrich. Calf thymus DNA was purchased from Beijing Biodee Biotechnology Co. Ltd. Thiazolyl blue tetrazolium bromide (M5655) was purchased from Sigma. Minimum Essential Media (MEM), Dulbecco's modified Eagle's medium (DMEM), fetal bovine serum (FBS), Dulbecco's phosphate buffered saline (PBS), trypsin–EDTA (0.5% trypsin, 5.3 mM EDTA tetra-sodium), and the antibiotic agents penicillin and streptomycin (100 U mL^–1^) were purchased from Life Technologies. Ultrapure water was obtained from a Millipore-Q system. All these commercially available reagents were used as received without further purification. BBR chloride was obtained from MERYER and purified by HPLC. Cucurbit[7]uril (CB7) was synthesized according to a literature method.^31^

### Instruments

UV-vis absorption spectra were taken on a Milton Roy Spectronic 3000 Array spectrophotometer. Photoluminescence (PL) spectra were recorded with a Perkin-Elmer (LS-55) fluorescence spectrometer. The fluorescence lifetime and absolute luminescence quantum yield were measured on an Edinburgh FLSP920 fluorescence spectrophotometer equipped with a xenon arc lamp (Xe900), a microsecond flash-lamp (uF900), a picosecond pulsed diode laser (EPL-375), a closed cycle cryostate (CS202*I-DMX-1SS, Advanced Research Systems) and an integrating sphere (0.1 nm step size, 0.3 second integration time, 5 repeats). The average particle size and size distribution of the samples were characterized on a Brookhaven ZetaPlus potential analyzer (Brookhaven Instruments Corporation, USA) at 25 °C. NMR spectra were recorded on a Bruker Advance DMX 400 spectrophotometer.

### Cell culturing

HeLa, A549 and MCF-10A cells were purchased from ATCC. 786-O cells containing the GFP gene were provided by Sun Yat-sen University Affiliated Cancer Hospital. All cell lines except HeLa cells were cultured in Dulbecco's modified Eagle's medium with 1% penicillin–streptomycin and 10% FBS, at 37 °C in a humidified incubator with 5% CO_2_. HeLa cells were cultured in MEM instead of DMEM. The culture medium was replaced every second day. By treating with 0.25% trypsin–EDTA solution, the cells were collected after they reached confluence.

### Cytotoxicity assay

HeLa, A549, and MCF-10A cells were seeded in 96-well plates at a density of 5000 cells per well, respectively. After 24 h cell culture, various concentrations of BBR chloride were added into the 96-well plate. After another 24 h cell culture, the medium was removed and the freshly prepared MTT medium solution (0.5 mg mL^–1^, 100 μL) was added into the 96-well plate. After incubation at 37 °C and 5% CO_2_ for 6 h, the MTT medium solution was removed carefully. After that, 100 μL DMSO was added into each well and the plate was gently shaken at room temperature to dissolve all the formed precipitates. A microplate reader was utilized to measure the absorbance at 570 nm from which the cell viability could be determined. Cell viability was expressed by the ratio of absorbance of the cells incubated with BBR chloride solution to that of the cells incubated with culture medium only.

### Cell imaging

For confocal microscopy imaging, cells were grown in a 35 mm Petri dish with a coverslip at 37 °C and 5% CO_2_. Firstly, cells were pre-treated with oleic acid (50 μM) for 6 h. Secondly, cells were incubated with BBR chloride (10 μM) and MeOTTMN (2 μM) for 30 min at 37 °C and 5% CO_2_. Then, the medium was removed and the cells were washed with PBS three times. After that, the cells were imaged using a confocal microscope (Zeiss laser scanning confocal microscope LSM7 DUO). For BBR chloride, the excitation wavelength was 488 nm and the emission filter was 500–580 nm; for MeOTTMN, the excitation wavelength was 488 nm and the emission filter was 600–744 nm.

### Photobleaching assay

HeLa cells stained with BBR chloride were irradiated with a 488 nm laser for 7.5 min continuously using a confocal microscope to evaluate BBR chloride's photostability. For comparison, 786-O renal carcinoma cells expressing green fluorescent protein (GFP) were irradiated under the same conditions as HeLa cells. Confocal images were captured every 15 s and parallelly compared to evaluate their photo-bleaching.

### Tissue imaging

Firstly, fresh mice liver tissue slices were cut to about 1 mm thickness. Secondly, liver slices were incubated with BBR chloride (20 μM) and MeOTTMN (4 μM) for 2 h at 37 °C and 5% CO_2_. Then, the medium was removed and the tissue slices were washed with PBS three times. After that, the tissue slices were imaged using a confocal microscope (Zeiss laser scanning confocal microscope LSM7 DUO). For BBR chloride, the excitation wavelength was 488 nm and the emission filter was 500–580 nm; for MeOTTMN, the excitation wavelength was 488 nm and the emission filter was 600–744 nm.

## Conflicts of interest

There are no conflicts to declare.

## Supplementary Material

Supplementary information
