# Supplementary material for: Exploration of biocompatible AIEgens from natural resources
Source: Chem Sci. 2018 Jun 29;9(31):6497–502. doi: 10.1039/c8sc01635f (PMC6115644; doi:10.1039/c8sc01635f)
Supplement: Supplementary file 1 [file SC-009-C8SC01635F-s001.pdf]

Electronic Supplementary Material (ESI) for Chemical Science.
This journal is © The Royal Society of Chemistry 2018

Electronic Supplementary Information

Exploration of Biocompatible AIEgens from Natural Resources

Yuan Gu,[ab#] Zheng Zhao,[ab#] Huifang Su,[ab] Pengfei Zhang,[ab] Junkai Liu,[c] Guangle Niu,[ab] Shiwu Li,[c] Zhaoyang Wang,[d] Ryan T. K. Kwok,[ab] Xin-Long Ni,[e] Jingzhi Sun,[d] Anjun Qin,[c] Jacky W. Y. Lam[ab] and Ben Zhong Tang[abcd]*

[a.] *Department of Chemistry, Department of Chemical and Biological Engineering, Hong Kong Branch of Chinese National Engineering Research Center for Tissue Restoration and Reconstruction, Institute for Advanced Study, Division of Biomedical Engineering, Division of Life Science and State Key Laboratory of Molecular Neuroscience, The Hong Kong University of Science and Technology (HKUST), Clear Water Bay, Kowloon, China. E-mail: tangbenz@ust.hk*

[b.] *HKUST-Shenzhen Research Institute, No. 9 Yuexing 1st RD, South Area Hi-tech Park, Nanshan, Shenzhen 518057 China.*

[c.] *NSFC Center for Luminescence from Molecular Aggregate, SCUT-HKUST Joint Research Laboratory, State Key Laboratory of Luminescent Materials and Devices, South China University of Technology, Guangzhou 510640, China.*

[d.] *MOE Key Laboratory of Macromolecular Synthesis and Functionalization, Department of Polymer Science and Engineering, Zhejiang University, Hangzhou 310027, China.*

[e.] *MOE Key Laboratory of Macrocyclic and Supramolecular Chemistry of Guizhou Province, Guizhou University, Guiyang, Guizhou 550025, China.*

[#.] *Y. Gu, Z. Zhao contributed equally to this work.*

^{1}H NMR of BBR Chloride

^{1}H NMR (400 MHz, DMSO, 25 °C), δ (ppm): 9.88 (s, 1H), 8.93 (s, 1H), 8.21–8.19 (d, *J* = 8.0 Hz, 1H), 8.01-7.99 (d, *J* = 8.0 Hz, 1H), 7.80 (s, 1H), 7.09 (s, 1H), 6.17 (s, 2H), 4.92-4.91 (d, *J* = 5.6 Hz, 2H), 4.09-4.07 (d, *J* = 8.0 Hz, 6H), 3.22-3.20 (m, 2H).

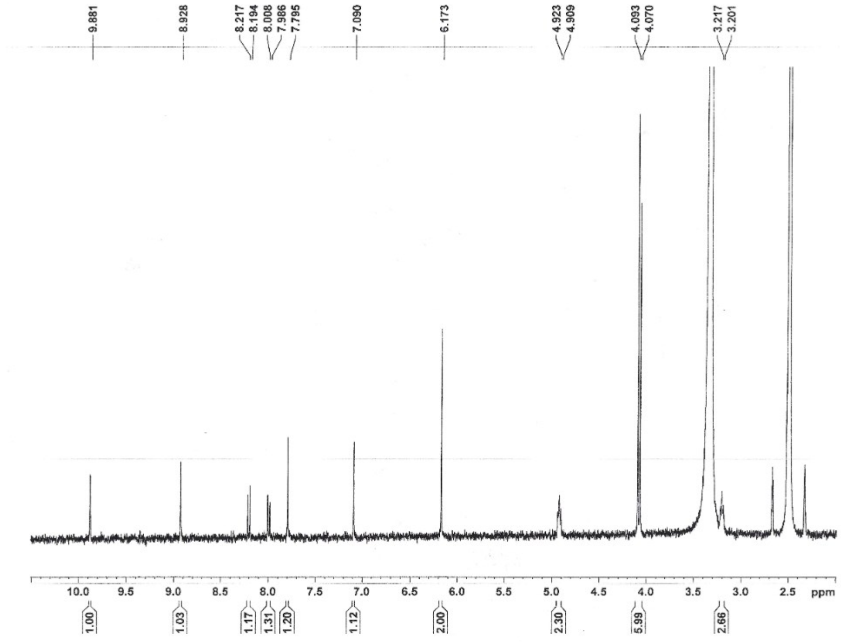

Fig. S1 ^{1}H NMR of BBR Chloride

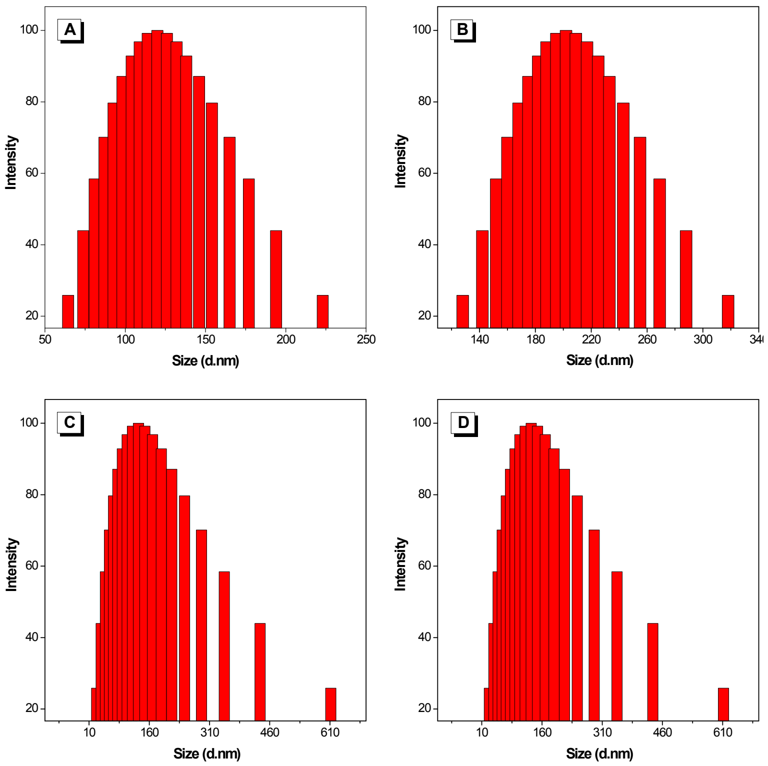

Fig. S2 DLS results of BBR Chloride in different THF/water mixtures: f_{THF} = 10% (A); f_{THF} = 50% (B); f_{THF} = 80% (C); f_{THF} = 99% (D). Concentration: 10μM.

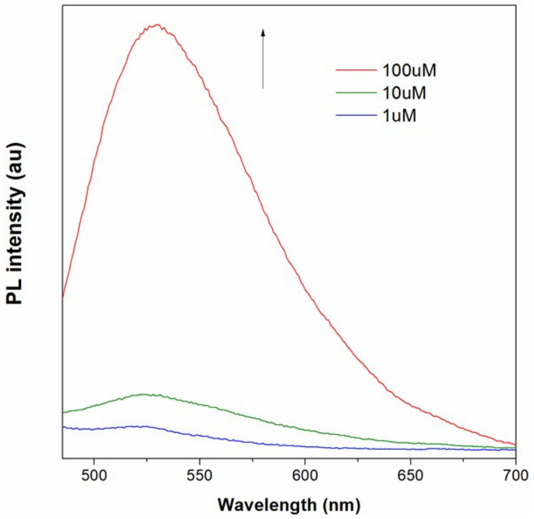

Fig. S3 Concentration dependence of the PL spectra of BBR Chloride in aqueous solution. Excitation wavelength: 405 nm. Slit width: 5 nm.

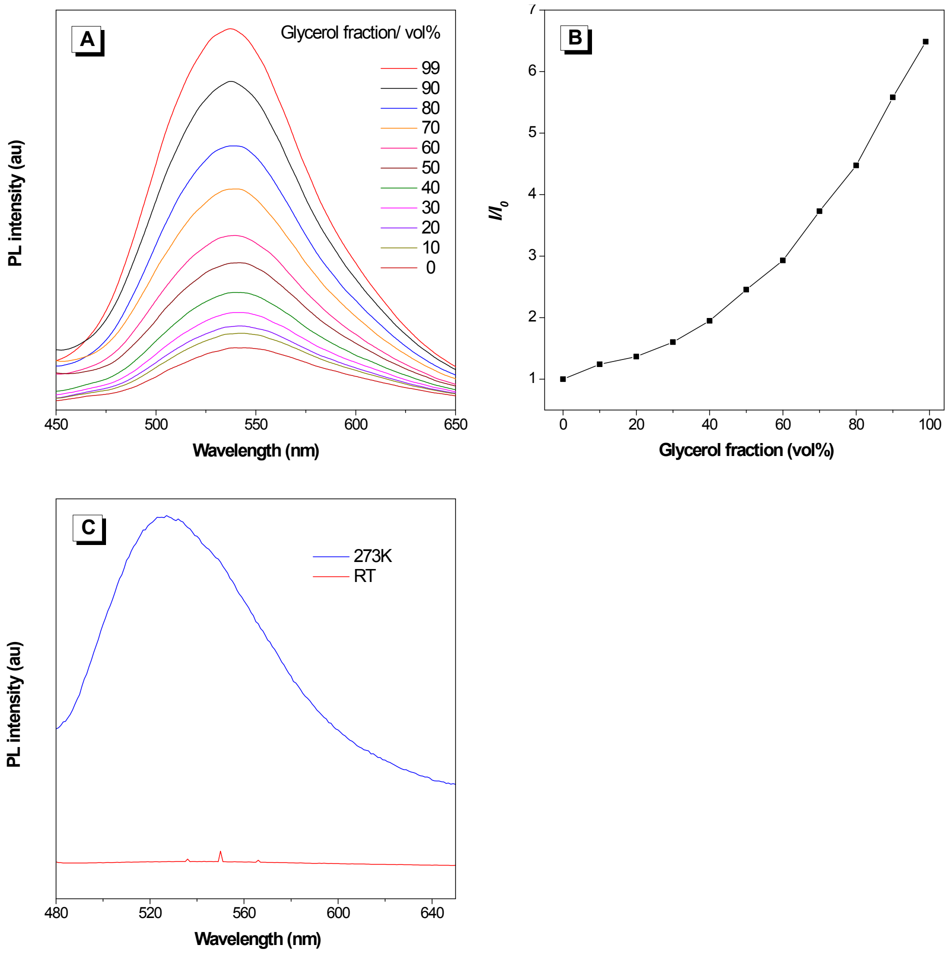

Fig. S4 (A) PL spectra of BBR Chloride in ethylene glycol/glycerol mixtures with different fractions of glycerol. Slit width: 15 nm. (B) Plot of the I/I_0 value of BBR Chloride versus the fractions of glycerol in the ethylene glycol/glycerol mixture. I_0 is the PL intensity of BBR Chloride in ethylene glycol. (C) Temperature dependence of the PL spectra of BBR Chloride in water solution. Slit width: 5 nm. Solution concentration: 10 μM; Excitation wavelength: 405 nm.

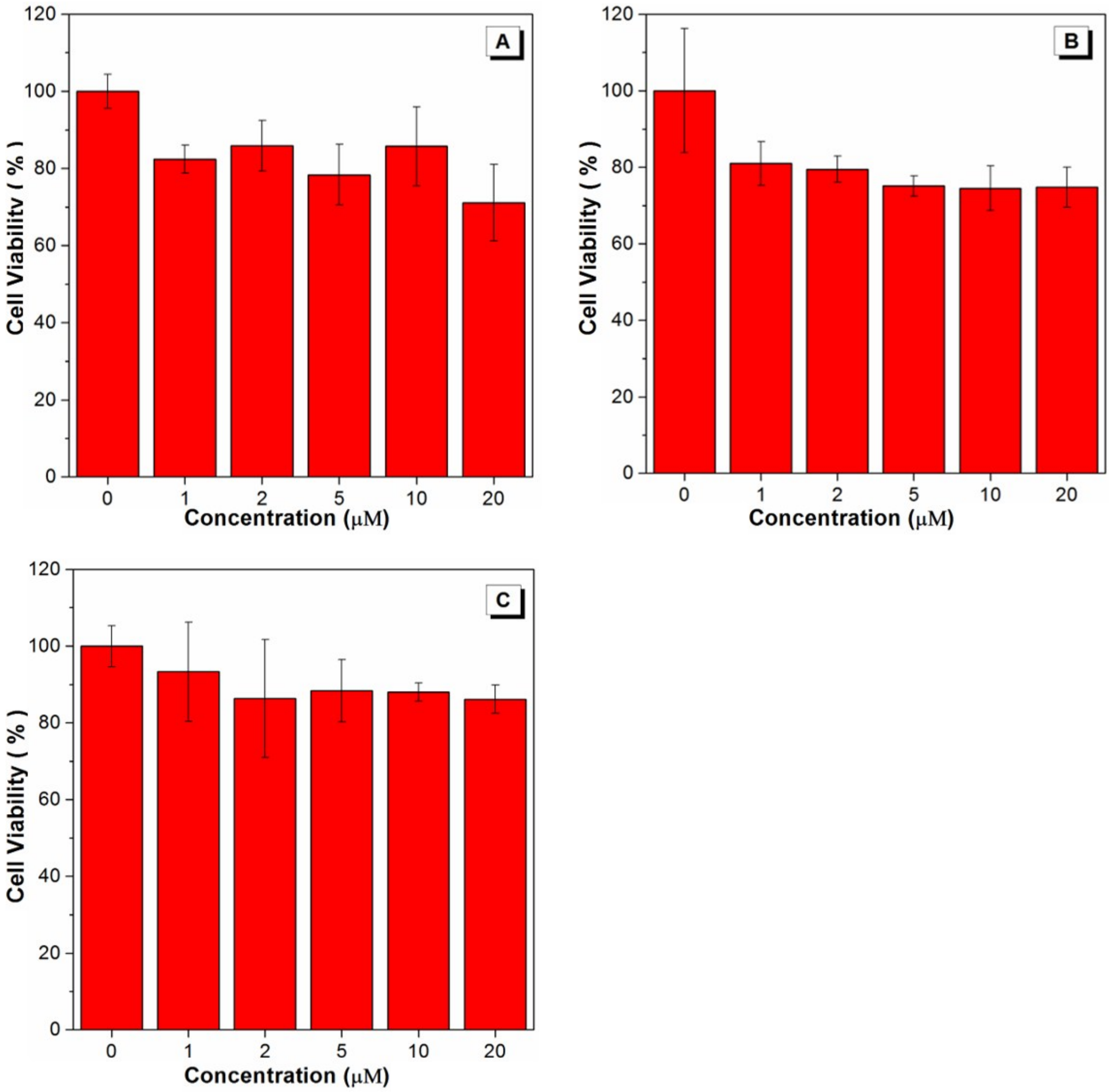

Fig. S5 Viability of HeLa cells (A), A549 cells (B), and MCF-10A cells (C) in the presence of different concentrations of BBR Chloride for 24 h. Data are expressed as mean value of six separate trials.

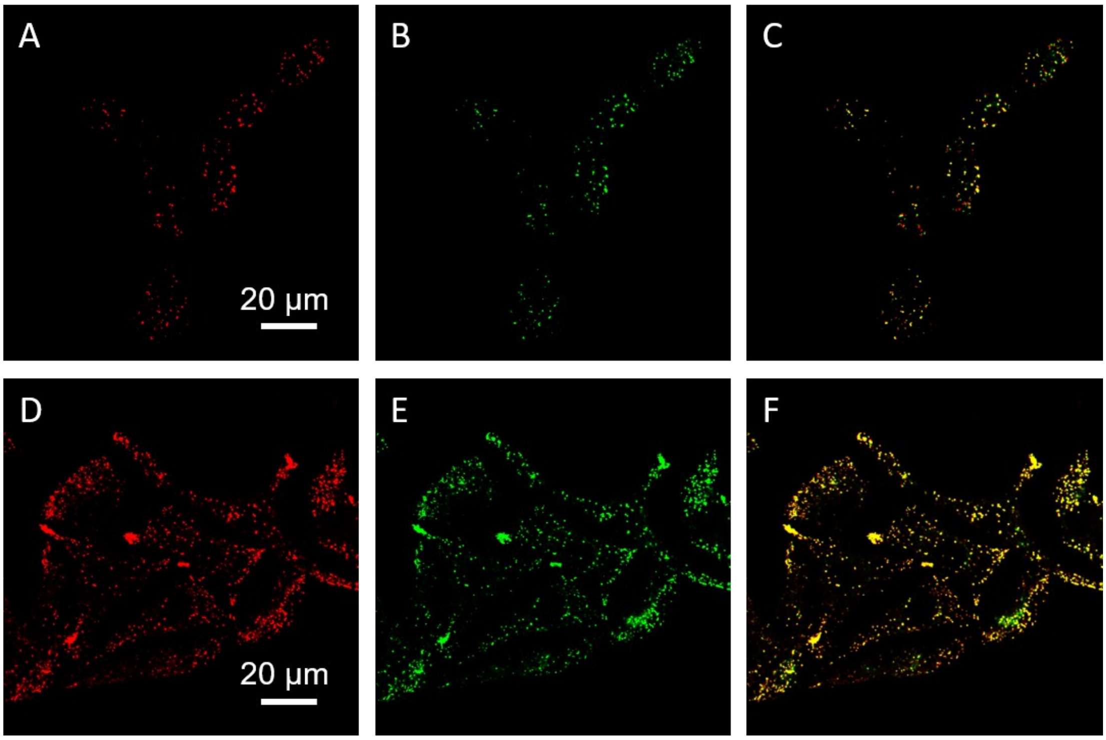

Fig. S6 (A–C) Confocal fluorescence images of A549 cells stained with MeOTTMN (A) and BBR Chloride (B), and the merged image of panel A and B (C). (D–F) Confocal fluorescence images of MCF-10A cells stained with MeOTTMN (D), BBR Chloride (E) and the merged image of panel D and E (F). λ_{ex}: 488 nm; scale bar = 20 µm.

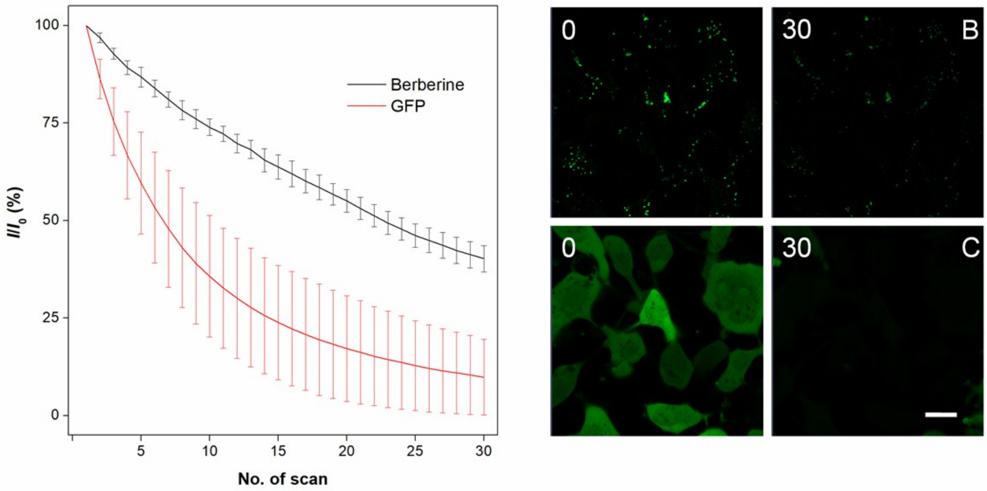

Fig. S7 (A) Photostability of BBR Chloride and green fluorescent protein (GFP) under continuous scanning at 488 nm (2.3 µW). I_0 is the initial PL intensity, while I is that of the corresponding sample after a designated No. of scan. (B and C) Confocal images of (B) Hela cells stained with BBR Chloride (10 µM) and (C) 786-O cells containing GFP gene before and after 30 scans of light irradiation. λ_{ex} : 488 nm; All the images share the same scale bar: 20 µm. Error bars are ± relative standard deviations (RSD), n = 6.

Table S1. Average Fluorescence Lifetimes and Radiative and Non-radiative Decay Rates for BBR Chloride in aqueous solution (10 μM), powder, and crystal.

Berberine Chloride	$<\tau>$ (ns)			k_r ($\times 10^8$ s^{-1})			k_{nr} ($\times 10^8$ s^{-1})		
	soln	crystal	powder	soln	crystal	powder	soln	crystal	powder
	0.68	7.93	4.86	0.022	0.19	0.24	14.69	1.08	1.82
